# Exploring the Relationship between Ovarian Cancer and Genital Microbiota: A Systematic Review and Meta-Analysis

**DOI:** 10.3390/jpm14040351

**Published:** 2024-03-27

**Authors:** Vito Andrea Capozzi, Giosuè Giordano Incognito, Elisa Scarpelli, Marco Palumbo, Cinzia Lucia Randazzo, Alessandra Pino, Marco La Verde, Carlo Ronsini, Gaetano Riemma, Michela Gaiano, Paola Romeo, Vittorio Palmara, Roberto Berretta, Stefano Cianci

**Affiliations:** 1 Department of Obstetrics and Gynecology, University Hospital of Parma, 43125 Parma, Italy; 2 Department of General Surgery and Medical Surgical Specialties, University of Catania, 95124 Catania, Italy; 3 Department of Agricultural, Food and Environment, University of Catania, Santa Sofia Street 100, 95123 Catania, Italy; 4 Department of Woman, Child and General and Specialized Surgery, University of Campania “Luigi Vanvitelli”, 80138 Naples, Italy; 5 Unit of Gynecology and Obstetrics, Department of Human Pathology of Adult and Childhood “G. Barresi”, University of Messina, 98122 Messina, Italy

**Keywords:** ovarian cancer, microbiota, cancerogenesis, gynecological cancer, gynecological microenvironment, ovarian malignancy

## Abstract

Ovarian cancer (OC) remains a significant health challenge globally, with high mortality rates despite advancements in treatment. Emerging research suggests a potential link between OC development and genital dysbiosis, implicating alterations in the microbiome composition as a contributing factor. To investigate this correlation, a meta-analysis was conducted following PRISMA and MOOSE guidelines, involving eight studies encompassing 3504 patients. Studies investigating the role of upper and inferior genital tract dysbiosis were included, with particular reference to HPV infection and/or history of pelvic inflammatory disease. The analysis revealed no significant difference in genital dysbiosis prevalence between OC patients and healthy controls. Although previous literature suggests associations between dysbiosis and gynecologic cancers, such as cervical and endometrial cancers, the findings regarding OC are inconclusive. Methodological variations and environmental factors may contribute to these discrepancies, underscoring the need for standardized methodologies and larger-scale studies. Despite the limitations, understanding the microbiome’s role in OC development holds promise for informing preventive and therapeutic strategies. A holistic approach to patient care, incorporating microbiome monitoring and personalized interventions, may offer insights into mitigating OC risk and improving treatment outcomes. Further research with robust methodologies is warranted to elucidate the complex interplay between dysbiosis and OC, potentially paving the way for novel preventive and therapeutic approaches.

## 1. Introduction

Ovarian cancer (OC) is the leading cause of gynecological malignancy-related mortality in high-income countries, due to its aggressive biological behavior and the lack of strategies for early diagnosis [1].

Despite significant advancements in treatment over the past decade, including the adoption of ultra-radical surgery and the availability of targeted therapies [2,3,4], the survival rate for OC is still poor, and it continues to rank as the fifth leading cause of cancer-related deaths among women [5]. Based on this, a crucial aspect in the fight against OC is the understanding of the physiopathology of the disease, and the role of different exogenous factors that can contribute to its initiation and progression. Known risk factors for OC include a positive family history of the disease, advanced age, the use of hormone replacement therapies, reproductive factors such as nulliparity and late menopause, as well as genetic mutations, most importantly BRCA1 and BRCA2 [6]. Other potential risk factors are endometriosis, obesity, and smoking, due to their role in enhancing a pro-inflammatory milieu [7].

In this context, one emerging area of research is the microbiome, which plays a crucial role in body homeostasis and has thus been investigated as a potentially significant oncogenic factor in various human cancers [8,9].

The normal physiological genital microenvironment is principally composed of Lactobacillus, characterized by one or more strains such as Iners, Crispatus, Gasseri, and Jensenii. These strains play a specific role in maintaining an acidic pH and displacing pathogen-binding sites [10,11].

Genital dysbiosis can be defined as a deviation from lactobacillus prevalence, with the presence of pathogens and anaerobic micro-organisms. This deviation can initiate a pathogenetic process that may vary based on the type of dysbiosis, anatomical area, and immune status [12].

The in-depth study of the human microbiome and the exogenous implementation of lactobacilli has enhanced our understanding of the microbiome’s importance in terms of maintaining body homeostasis, protecting against external agents, and supporting immune defense [13,14].

The human microbiome composition, as previously demonstrated, could be different based on anatomical district, age, race, diet, and body composition. The Microbiota Project revealed the body-site specificity of microbiota composition and function [15]. Moreover, alterations in the microbiome within specific regions can certainly be associated with a diverse range of diseases, from infections to disruptions in organ function [14]. 

The possible interplay between site-specific dysbiosis, history of infectious diseases, and cancer development is still unclear. While some evidence from the literature has been reported, the available data are not consistent. In the context of OC, this potential relationship is even more debated, as OC etiology can be attributed to multifactorial agents, including genetic predisposition, family history, and hormonal status [16,17].

The rationale of oncogenesis and genital dysbiosis is founded on the mechanism of chronic inflammation and oxidative stress, leading to genotoxicity and genomic alterations [18]. 

The present study aims to report the available evidence regarding OC and genital microbiome alterations. By doing so, we aim to clarify the relationship between OC development and dysbiosis in the upper and lower genital tracts, investigating its potential as a contributing factor to the disease’s etiology.

## 2. Materials and Methods

The systematic review was registered in the PROSPERO database with registration number CRD42024517796 to ensure transparency and adherence to best methodological practices, as recommended by international standards. The research strategy was decided a priori, following the Preferred Reporting Items for Systematic Reviews and Meta-Analyses (PRISMA) [19] and the Meta-analysis of Observational Studies in Epidemiology (MOOSE) statement guidelines (supplement M1) [20], defining the methodology for the literature search, article evaluations, and inclusion criteria. Then the data analysis was performed.

**Figure 1:**
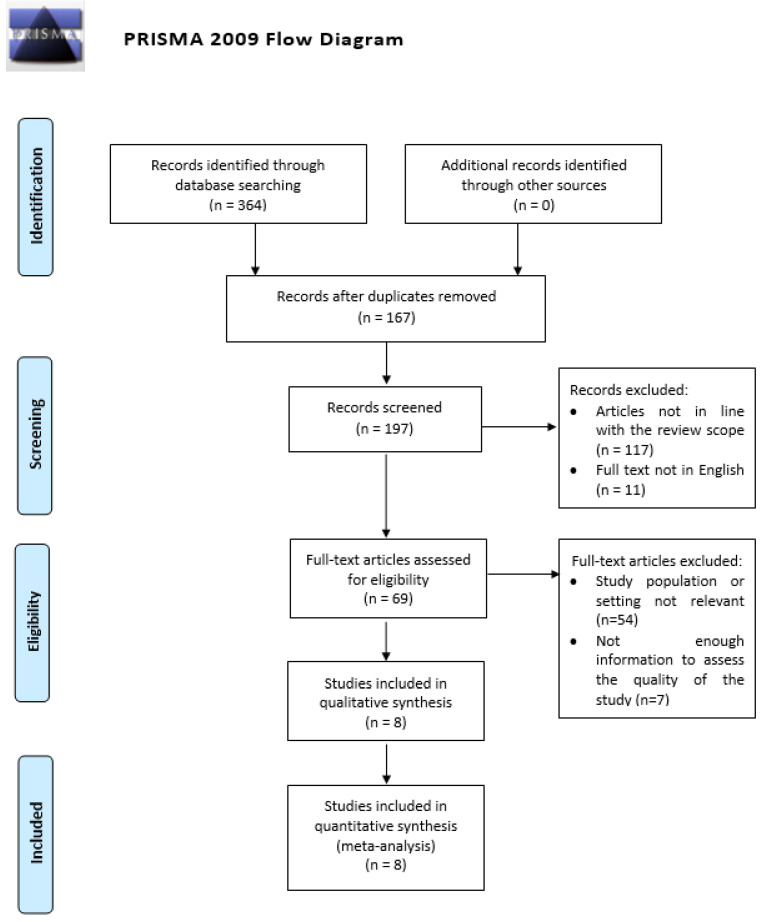
PRISMA flow diagram [21].

The literature search was performed using PubMed, Scopus, and Cochrane databases, evaluating the available articles until September 2023. We extracted all the articles with a combination of the following keywords and medical subject headings (MeSHs): ovarian cancer and microbiome; dysbiosis and ovarian cancer; microbiota and ovarian cancer; and microbes and ovarian cancer. Geographic restrictions were not applied. The PRISMA flow diagram (Figure 1) summarizes the search strategy. 

All studies evaluating the genital microbiome of patients affected by OC compared with healthy patients have been included in the final analysis, while commentaries, editorials, reviews, and abstracts have been excluded. 

Two authors (G.G.I. and C.M.) reviewed and selected all abstracts independently. The relevance of each article was determined by the agreement of both reviewers. Based on the aim of our study, the authors extracted the full text of selected articles and selected the available data. In case of discrepancy between the two researchers, a third senior author (S.C.) was called to make the final decision. 

Two other authors (F.A.G. and V.A.C.) were responsible for evaluating and reviewing the selected articles and their eventual bias. The risk of bias was assessed by the two authors using the ROBINS-E tool [22,23]. In case of discrepancies, a third author (ES) contributed to the final evaluation, which was established through agreement with the two authors. Potential publication bias was assessed using the Egger’s test [24] and trim-and-fill analysis was conducted to adjust for any detected bias [25].

The primary endpoint of the meta-analysis was to assess the evaluation of an eventual relationship between genital dysbiosis and OC. 

### Statistical Analysis

The statistical analysis aimed to assess differences in the reported incidence of genital dysbiosis between OC patients and healthy controls. Categorical variables were presented as numbers and percentages, while Cohen’s effect size was employed to measure the magnitude of differences for continuous variables [26].

To gauge the heterogeneity among the included studies, we utilized the I^2^ test, where an I^2^ value exceeding 50% indicated substantial heterogeneity across the studies [27]. Regardless of the I^2^ test result, we opted for a random-effects model to accommodate potential variations among the studies.

Statistical significance was determined with a threshold of *p* < 0.05. All analyses were conducted using Prometa Software version 3.0.0.

## 3. Results

**Figure 2:**
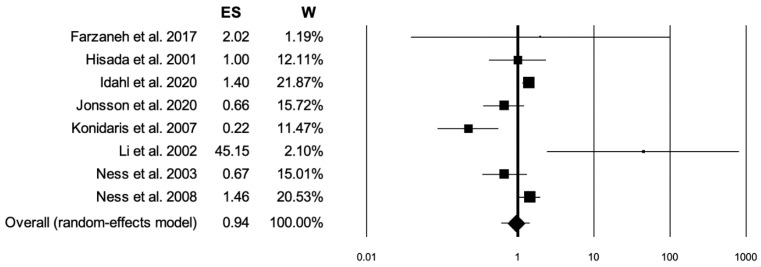
Forest plot: ES effect size; W weight [15,16,17,18,19,20,22,23].

Of the 364 studies initially identified, 8 studies met the inclusion criteria (Table 1) [28,29,30,31,32,33,34,35]. Of the 4300 patients, 1421 had OC (Group 1) and 2879 were healthy patients (Group 2). The number of patients and instances of dysbiosis for each study is summarized in Table 2. A total of 670 (40.2%) instances of dysbiosis was reported in the OC group, with 573 (31.2%) instances in the healthy patients group. The meta-analysis revealed no significant difference in dysbiosis between the two groups, with a *p*-value of 0.779 and an effect size of 0.94 (95% confidence interval 0.61–1.47), as illustrated in the forest plot (Figure 2). The heterogeneity analysis for studies included showed an I^2^ value of 76.04, *p* < 0.001. 

**Figure 3:**
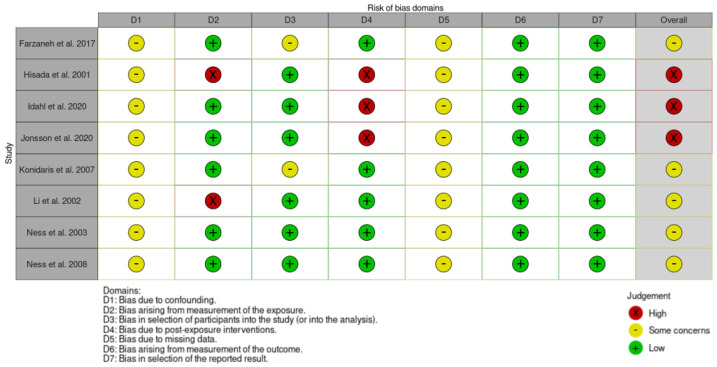
Risk-of-bias assessment according to ROBIN-E tool [15,16,17,18,19,20,22,23].

According to the risk-of-bias assessment using the ROBINS-E tool, overall three studies were judged at high risk of bias, because of the lack of information and possible deviation from the exposure between the moment of dysbiosis evaluation and the occurrence of the outcome defined as OC occurrence [29,30,31]. Moreover, none of the studies reported adjustments for confounding factors. The risk-of-bias assessment summary is reported in Figure 3.

**Figure 4:**
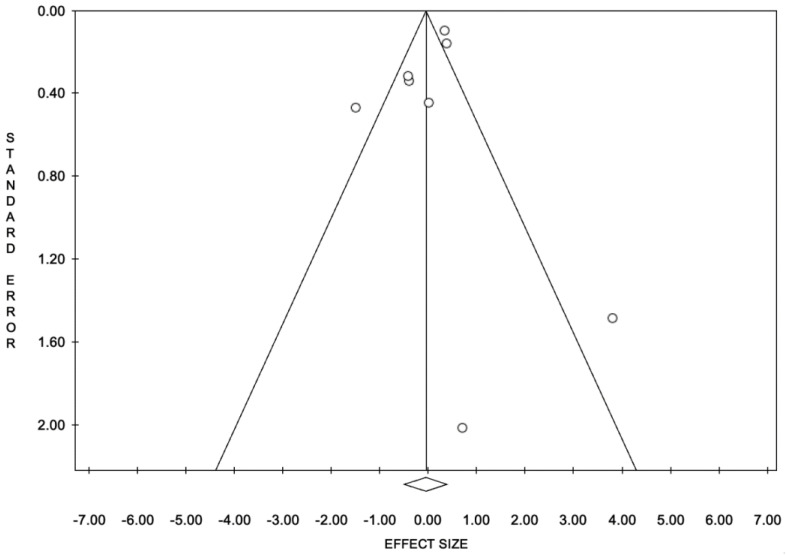
Trim-and-fill funnel plot for publication bias.

The publication bias through trim-and-fill analysis showed that no study was trimmed (Figure 4).

### 3.1. Results—History of Chlamydia Infection

Among the articles included in our metanalysis, four manuscripts investigated the interplay between a history of chlamydia infection and the occurrence of OC [30,31,34,35].

Ness et al. [34] in 2003 conducted a population-based case-control analysis on 117 women with OC and 171 age and ethnicity-matched control subjects. All patients underwent measurements of Chlamydia Trachomatis antibodies and chlamydial heat shock proteins, using an ELISA assay. 

The same author [35] published a further case series in 2008 on a larger population (521 vs. 766), employing the same serologic assays. However, the results were inconsistent in showing any link between previous chlamydia infection and OC occurrence.

In a case-control study on a population derived from the EPIC study (European Prospective Investigation into Cancer and Nutrition) by Idahl et al. [30], an assay of serum antibodies against a larger panel of possible pathogens was utilized including Chlamydia trachomatis, Mycoplasma Genitalium, herpes simplex 2, and HPV. In total, 791 cases and 1669 matched controls were included. None of the pathogens revealed an association with OC development.

Consistent with previous publications, Jonsson et al. [31] reported similar observations from a prospective nested case-control analysis of 92 high-grade serous OC patients vs. 359 matched controls. Again, the authors investigated serology of past infection by Chlamydia trachomatis using ELISA immunoassay, without evidence of significant correlation.

### 3.2. Results—HPV and Ovarian Cancer

Four of the included articles explored the potential association between HPV infection and OC incidence.

Farazaneh et al. [28] investigated the presence of the HPV virus in 105 patients diagnosed with ovarian tumors, through DNA extractions and PCR amplification. The virus was not detected in any of the ovarian tissue samples.

Similarly, Konidaris et al. [32] reported the presence of HPV in specimens, in a population of 43 patients with malignancy and 84 patients with benign gynecologic tumors. In this case, in situ hybridization was used for HPV 16, 18, 31, 33, and 51. An incidence of 27.9% vs. 45.2% of HPV positivity was in cases vs. controls, without significant difference (p 0.2).

Hisada et al. [29] investigated the role of HPV infection in the development of gynecological cancers, reporting the seropositivity for HPV 16 in 152 with gynecological cancers and 172 matched controls. Among them, 36 patients had a diagnosis of OC, and HPV positivity was comparable in patients and matched controls (22%, *p* = 0.9).

In conclusion, Li et al. explored the link between p53 polymorphism and HPV-associated carcinogenesis [33]. In doing so, they also examined the incidence of HPV 16 and 18 in 39 OC patients through in situ hybridization and PCR on surgical specimens, which appeared significantly higher compared to 50 healthy patients.

## 4. Discussion

### 4.1. Main Findings

OC represents a significant challenge in gynecological oncology due to its high mortality rates despite advancements in treatment options. The understanding of etiologic patterns and hypotheses concerning its pathogenesis remains largely unknown. Our investigation aimed to explore the potential relationship between OC and alterations in the genital microbiome. We conducted a comprehensive meta-analysis of available literature, identifying eight relevant studies. Our analysis encompassed a total of 4300 patients, including 1421 OC cases and 2879 healthy controls. Surprisingly, our findings did not reveal a significant difference in genital dysbiosis between OC patients and healthy individuals (*p* = 0.779). Subgroup analyses focusing on the history of chlamydia infection and the association of human papillomavirus (HPV) with OC incidence provided mixed results. While some studies suggested a potential link, others failed to establish significant correlations. For instance, Farazaneh et al. [18] and Konidaris et al. [23] reported conflicting findings regarding HPV presence in ovarian tumors, while Hisada et al. [19] found comparable HPV seropositivity rates between OC patients and controls. Conversely, Li et al. [24] demonstrated significantly higher incidence rates of HPV 16 and 18 in OC patients compared to healthy individuals. Overall, our meta-analysis highlights the complex interplay between microbiome alterations and OC pathogenesis, suggesting that further research is needed to elucidate these relationships fully and understand their clinical implications.

### 4.2. Results in the Context of Published Literature

#### 4.2.1. Microbiota and Cancer

The microbiota and its importance in the maintenance of body homeostasis is well known. The composition of the microbiota can vary, exhibiting distinct functions depending on the specific body regions, such as the skin, bowel, or natural orifices [36]. One of the most investigated is the gut microbiome, as its function has been reported to be related not only to bowel function, but also to neurological, psychological, and systemic functions [37]. Gut dysbiosis was even associated not only with colon cancer, but also with other cancer types, including OC [38,39].

The interplay between microorganisms and cancer development is not a novelty in oncology. Past research has shown that disrupting the microbial homeostasis of organs can trigger immune activation, leading to a persistent state of inflammation. This, in turn, can activate pro-oncogenic factors such as tumor necrosis factor (TNF)-α, interleukin (IL)-6, and vascular endothelial growth factor (VEGF). Prolonged exposure to these factors may increase the risk of carcinogenesis [40]. 

#### 4.2.2. Microbiota and Gynecological Malignancies

The impact of microbiota on carcinogenesis encompasses two key aspects: first, the influence of genomic contributions of gut microbiota on various pathologic and physiopathologic conditions throughout the entire organism; and second, as previously mentioned, the impact of site-specific dysbiosis on organ-specific function and physiology.

Regarding the female genital tract, recent findings have identified not only a vaginal microbiota, also known as lower tract microbiota, but also an upper genital tract microbiota. This upper genital tract microbiota appears to differ in composition, exhibiting greater biodiversity primarily due to the lower representation of Lactobacillus species.

Recent investigations explored the intricate link between genital microbiota and carcinogenesis. Previous studies have suggested that the transition from HPV infection to cervical cancer (CC) may be associated with non-Lactobacillus pathogens. These pathogens could impede antibody clearance, thereby allowing the persistence of the HPV virus and subsequent carcinogenesis [41,42]. These studies revealed that the depletion of Lactobacillus spp. and the presence of a multispecies microbial vaginal composition were significantly associated with patients affected by cervical dysplasia compared to healthy controls [43]. Furthermore, additional studies identified specific bacteria more frequently detected in patients with cervical disease, including Atopobium, Prevotella, Gardnerella, Peptostreptococcus, and Anaerococcus [44]. 

Other hypotheses propose the possible interaction mechanism represented by the direct cellular damage caused by pathogens, such as anti-apoptosis, chronic inflammation, and angiogenesis, contributing with HPV to the development of cellular alterations [44,45].

Even for endometrial cancer (EC), some evidence suggests that microbiota alteration could be a risk factor for EC development. Considering well-recognized risk factors such as obesity, menopausal state, genetic factors, and metabolic disorders, it is noteworthy that all these factors are also applicable to gut and genital dysbiosis [46,47]. 

Some studies investigated the endometrial microbial composition of patients affected by endometrial hyperplasia compared with healthy controls; they found characteristic microbial patterns more pronounced in the study group compared to controls. The authors suggested the possibility of dysbiosis playing a role in chronic endometrial inflammation and low pH, promoting cellular damage and increasing the risk of cancer development [48,49]. 

#### 4.2.3. Microbiota and Ovarian Cancer

When examining the complex relationship between OC and the microbiota, the existing literature provides some evidence, albeit within the confines of studies that examined a relatively small number of patients. 

##### Pelvic Inflammatory Disease and Ovarian Cancer

Pelvic inflammatory disease, a condition characterized by inflammation of pelvic organs such as the uterus, fallopian tubes, and ovaries, has been explored as a potential risk factor for OC development. According to epidemiological studies, the self-reported incidence of PID is around 4% in women aged 18–44 years; however, this figure is likely to be underestimated due to the variability in symptom presentation and the social stigma often associated with it [50]. Chlamydia trachomatis is one of the primary pathogens associated with pelvic inflammatory disease (PID) in women, with other common pathogens including Neisseria gonorrhoeae and Mycoplasma genitalium [51,52].

Genital dysbiosis, characterized by an imbalance in microbial flora in the genital region, has been associated with an increased risk of developing PID, underscoring the importance of maintaining a balanced microbial environment for women’s reproductive health [53].

One of the rationales behind hypothesizing a link between pelvic inflammatory disease (PID) and ovarian tumors is the inflammatory state induced by tubal infections on the tubal epithelium. Chronic infections affecting the fallopian tubes and ovaries might elevate the risk of OC development by fostering the activation of pro-oncogenic mediators [54]. Indeed, it is now recognized that high-grade serous ovarian tumors, constituting the most prevalent histotype, originate from the tubal epithelium [55].

Research indicates several potential molecular and inflammatory mechanisms underlying this connection. Persistent activation of inflammatory pathways due to chronic inflammation, a hallmark of PID, may result in the release of pro-inflammatory cytokines and chemokines. These molecules can establish a microenvironment favorable for tumor initiation, promotion, and progression [56]. Moreover, chronic inflammation could induce DNA damage through the generation of reactive oxygen species (ROS) and activation of DNA-damaging enzymes, contributing to the accumulation of genetic mutations associated with OC development [57].

Additionally, dysbiosis, characterized by alterations in the composition of genital microbiota often observed in PID, could exacerbate inflammatory responses and promote tumorigenesis. Dysbiotic microbial communities might produce metabolites and toxins, leading to local inflammation and tissue damage, thus fostering an environment conducive to oncogenesis. Furthermore, dysbiosis-induced changes in the host immune response may compromise immune surveillance against emerging tumor cells, further fostering OC development [58].

Epidemiological studies have furnished evidence supporting the correlation between PID and OC risk [59,60]; however, the consistency of these observations is questionable. Our meta-analysis does not support a conclusive link between dysbiosis and OC, particularly concerning prior or ongoing genital infections. However, it is essential to consider that these findings might stem from the heterogeneity in the pathogens considered and the diverse diagnostic methods employed across the studies.

Specifically addressing the association between chlamydia infection and OC, the comparison of the included studies presents limitations due to the heterogeneity in antibody types measured, assay methods, and sample collection procedures. Moreover, the timing of the antibody analysis concerning OC diagnosis and treatment may influence the antibody levels and, consequently, the observed associations [30,31,34,35].

##### HPV and Ovarian Cancer

HPV infection has long been recognized as a major risk factor for various cancers, particularly cervical cancer [61]. However, its potential role in the development of OC has been a subject of debate and investigation. Numerous studies have sought to explore the association between HPV infection and OC, driven by the hypothesis that HPV may exhibit tropism for ovarian and tubal epithelial cells. Epidemiological investigations have examined the prevalence of HPV infection in OC patients compared to healthy controls, aiming to establish a potential causal link between HPV and OC development. However, the findings from these studies have been inconsistent and inconclusive.

Several plausible mechanisms have been proposed to explain how HPV infection could contribute to ovarian carcinogenesis. HPV may directly infect ovarian epithelial cells, leading to cellular transformation and tumor initiation [62]. Additionally, HPV-induced immune dysregulation and inflammation could create a microenvironment conducive to oncogenesis and tumor progression in the ovaries [63].

Furthermore, the potential role of HPV oncoproteins, particularly E6 and E7, in disrupting cellular regulatory pathways and promoting genomic instability has been implicated in ovarian tumorigenesis [64]. These viral proteins may interact with host cell proteins involved in cell cycle regulation, apoptosis, and DNA repair, ultimately facilitating malignant transformation in HPV-infected ovarian cells [65].

Interestingly, even more complex interactions between HPV and host cells have been reported: some evidence exists regarding the ability of HPV-related oncoproteins E6 and E7 to inhibit the activity of tumor suppressor genes such as p53, potentially playing a crucial role in favoring carcinogenesis and tumor progression [66,67].

In a systematic review conducted by Rosa et al., which included data from multiple observational studies, a high prevalence of HPV was found in women with OC, although its potential pathogenetic role was not assessed [68].

In line with these observations, our analysis does not reveal a significant association between HPV positivity and ovarian tumors. While none of the examined studies demonstrated a significant association between HPV positivity and ovarian tumors, the heterogeneity in analysis methods, sampling locations, and analyzed genotypes prevents definitive conclusions being drawn from these studies [28,29,31,32].

The complexity of the relationship between HPV and OC is further compounded by factors such as the variability in analysis methods, sampling locations, and analyzed genotypes across different studies. Additionally, the timing of HPV infection analysis relative to the diagnosis and treatment of ovarian tumors introduces an additional layer of intricacy. Environmental changes during cancer treatment and progression may also influence the composition of the microbiota, further complicating the relationship between HPV infection and OC [35].

Moreover, the biological mechanisms underlying any potential association between HPV infection and OC remain poorly understood. It is unclear whether HPV infection may directly contribute to oncogenesis in ovarian epithelial cells or if other factors potentially associated with HPV infection and persistence, such as immune dysregulation or chronic inflammation, play a role in the carcinogenic process. Indeed, the presence and persistence of HPV have been associated with alterations in the vaginal microbiome, and most importantly, bacterial vaginosis due to impaired Lactobacillus function [69,70]. In this context, the exogenous implementation of Lactobacillus crispatus has been proposed as a preventive strategy [71].

### 4.3. Strengths and Limitations

The present study demonstrates several strengths, including its comprehensive literature review encompassing a meta-analysis methodology, as well as adherence to PRISMA and MOOSE guidelines. Additionally, the study’s focus on exploring the potential relationship between OC and alterations in the genital microbiome addresses a significant gap in current research, offering valuable insights into a relatively understudied area.

However, several limitations must be acknowledged. Methodological heterogeneity across studies represents a notable challenge, potentially influencing the robustness and generalizability of our findings. The diverse diagnostic methods employed and the variability in sample collection procedures may introduce biases and inconsistencies in the data, impacting the overall interpretation of results. Moreover, the relatively limited sample size across included studies could affect the statistical power and precision of our analysis, potentially limiting the strength of our conclusions.

Furthermore, the complexity of the microbiota–cancer relationship poses inherent challenges in establishing definitive associations. Environmental factors, including treatment interventions and disease progression, may confound the observed relationships, necessitating careful consideration and interpretation of results. Additionally, the diverse microbial compositions and pathogen-specific responses further complicate the analysis, highlighting the need for more standardized methodologies and larger-scale studies to elucidate these intricate relationships conclusively.

In conclusion, while our study provides valuable insights into the potential link between OC and alterations in the genital microbiome, further research incorporating larger sample sizes, standardized methodologies, and longitudinal assessments is warranted to validate and expand upon our findings.

### 4.4. Implications for Practice and Future Directions

Understanding the potential link between microbiota and OC development holds promise for guiding preventive and therapeutic strategies.

Firstly, the recognition of the microbiome’s influence on cancer development, as evidenced in other gynecologic malignancies like cervical and endometrial cancers, underscores the need for a holistic approach to patient care. This may involve monitoring microbial compositions, implementing interventions to restore or maintain a healthy microbiome, and considering microbiome-related factors in the development of personalized treatment plans. It is noteworthy that within the context of OC, there is some evidence in the literature supporting an association between microbiota composition, disease stage, and response to treatment. Studies have elucidated a distinct OC microbiome characterized by specific microbial taxa enrichment, which correlates with disease stage and treatment outcomes [72]. These findings underscore the potential of microbiota-based markers in facilitating early detection and predicting treatment response in ovarian cancer patients. Moreover, experiments on patients-derived organoids have replicated an inflammatory response to genital dysbiosis with potential impact on carcinogenesis induction [73].

The lack of a definitive association between dysbiosis and ovarian cancer in our meta-analysis, despite encouraging evidence in other pathologies and preclinical studies on ovarian cancer, underscores the necessity for ongoing research and the refinement of study methodologies. While our findings provide valuable insights, further investigations with larger sample sizes and standardized methodologies are warranted to elucidate the complex relationships between microbiota and OC pathogenesis conclusively. Clinicians and researchers should collaborate to conduct prospective studies that explore longitudinal changes in the microbiome throughout the course of OC development and treatment. By addressing these knowledge gaps, clinicians can better tailor preventive and therapeutic interventions to mitigate OC risk and improve patient outcomes.

## 5. Conclusions

In conclusion, despite methodological variations, our meta-analysis did not reveal a significant dysbiosis difference between OC patients and healthy controls. The inconclusive nature of our findings underscores the complexity of the OC–microbiome relationship, highlighting the need for standardized methodologies in sample processing, sequencing techniques, and data analysis to ensure comparability and reproducibility across studies. Furthermore, we emphasize the importance of considering the time between exposure to dysbiosis and the onset of ovarian tumor development, along with confounding variables that may influence the microbiome–host interaction in OC. Future research efforts should prioritize the implementation of consistent methodologies to unravel the intricate interplay between the microbiota and OC. This ongoing investigation holds promise for informing potential preventive and therapeutic strategies for OC, providing valuable insights into the role of dysbiosis in the pathogenesis of this complex disease.

## Figures and Tables

**Table 1:** Articles included in the meta-analysis: type and timing of infection analysis.

Authors	Design of the Study	Country	Population	Type of Analysis	Timing of the Analysis vs. Ovarian Cancer Diagnosis
Farzaneh et al., 2017 [15]	Cross-sectional study	Iran	Patients with epithelial benign and malignant ovarian tumors	HPV DNA extraction from paraffin-embedded blocks and PCR	HPV presence in tumor tissue
Hisada et al., 2001 [16]	Retrospective analysis from prospective cohort study	USA	Pregnant women	ELISA assay for HPV 16 on serum samples	Serum samples collected at the time of enrollment (pregnancy), follow up for cancer occurrence
Idahl et al., 2020 [17]	Nested case-control study from prospective cohort study	Europe (international study)	Women with positive vs. negative serology for STI	Serum antibodies against Chlamydia trachomatis, Mycoplasma genitalium, herpes simplex virus type 2 (HSV-2) and human papillomavirus (HPV) 16, 18, and 45 were assessed using multiplex fluorescent bead-based serology assay	Serum samples collected at the time of enrollment (healthy patients), follow up for cancer occurrence
Jonsson et al., 2020 [18]	Nested case-control study from prospective cohort study	Sweden	Women with positive vs. negative serology for Chlamydia Trachomatis	Plasma C. trachomatis IgG analyzed using micro- immunofluorescence test; chlamydial Heat Shock Protein 60 IgG (cHSP60) and anti-MUC1 IgG analyzed with ELISA technique	Serum samples collected at the time of enrollment (healthy patients), follow up for cancer occurrence
Konidaris et al., 2007 [19]	Prospective nonrandomized study	Greece	Patients with epithelial benign and malignant ovarian tumors	Seven oncogenic types of HPV (6, 11 16, 18, 31, 33, and 51) using the in situ hybridization technique on specimens	HPV presence in tumor tissue
Li et al., 2002 [20]	Case-control study	China	Ovarian cancer patients vs. healthy controls	HPV 16 and 18 presence through in situ hybridization and PCR on surgical specimens vs. HPV DNA extraction from blood samples of healthy patients	HPV presence in tumor tissue
Ness et al., 2003 [22]	Population-based case-control study	Hawaii	Ovarian cancer patients vs. healthy controls	Serologic ELISA assay for the detection of antibodies to Chlamydia trachomatis, to chlamydial heat shock protein (CHSP) 60, and to CHSP10	Serum samples from OC patients
Ness et al., 2008 [23]	Population-based case-control study	USA	Ovarian cancer patients vs. healthy controls	Serologic ELISA assay for the detection of antibodies to Chlamydia trachomatis, to chlamydial heat shock protein (CHSP) 60, and to CHSP10	Serum samples from OC patients

OC ovarian cancer; STI sexually transmitted infections.

**Table 2:** Articles included in the metanalysis: incidence of dysbiosis.

Authors	Patients	Dysbiosis in Ovarian Cancer Patients (%)	Dysbiosis in Healthy Patients (%)	*p*-Value
Farzaneh et al., 2017 [15]	105	0.0	0.0	0.727
Hisada et al., 2001 [16]	230	22.2	22.2	0.933
Idahl et al., 2020 [17]	1723	41.0	33.2	0.001
Jonsson et al., 2020 [18]	451	16.3	22.8	0.187
Konidaris et al., 2007 [19]	127	27.9	45.2	0.200
Li et al., 2002 [20]	89	66.7	0.0	0.010
Ness et al., 2003 [22]	288	31.6	40.7	0.245
Ness et al., 2008 [23]	1287	47.6	38.4	0.017

## Data Availability

The data that support the findings of this study are available on request from the corresponding author (ES).
